# HIV patients retention and attrition in care and their determinants in Ethiopia: a systematic review and meta-analysis

**DOI:** 10.1186/s12879-020-05168-3

**Published:** 2020-06-22

**Authors:** Nurilign Abebe Moges, Adesina Olubukola, Okunlola Micheal, Yemane Berhane

**Affiliations:** 1 Department of Public Health, College of Health Sciences, Debre Markos University, Debre Markos, Ethiopia; 2 Pan African University, Life and Earth Sciences Including Health and Agriculture Institute (PAULESI), University of Ibadan, Ibadan, Nigeria; 3 Department of Obstetrics and Gynecology, University College Hospital, University of Ibadan, Ibadan, Nigeria; 4 Department of Epidemiology, Addis Continental Institute of Public Health, Addis Ababa, Ethiopia

**Keywords:** Retention, Attrition, HIV patient, Systematic review, Ethiopia

## Abstract

**Background:**

There is paucity of evidence on the magnitude of HIV patients’ retention and attrition in Ethiopia. Hence, the aim of this study was to determine the pooled magnitude of HIV patient clinical retention and attrition and to identify factors associated with retention and attrition in Ethiopia.

**Methods:**

Systematic review and meta-analysis were done among studies conducted in Ethiopia using the Preferred Reporting Items for Systematic Reviews and Meta-Analyses (PRISMA) guideline. Both published and unpublished studies conducted from January 1, 2005 to June 6th, 2019 were included. Major databases and search engines such as Google Scholar, PUBMED, African Journals Online (AJOL) and unpublished sources were searched to retrieve relevant articles. Data were assessed for quality, heterogeneity and publication bias. Analysis was conducted using STATA version 14 software.

**Result:**

From a total of 45 studies 546,250 study participants were included in this review. The pooled magnitude of retention in care among HIV patients was 70.65% (95% CI, 68.19, 73.11). The overall magnitude of loss to follow up 15.17% (95% CI, 11.86, 18.47), transfer out 11.17% (95% CI, 7.12, 15.21) and death rate were 6.75% (95% CI, 6.22, 7.27). Major determinants of attrition were being unmarried patient (OR 1.52, 95% CI: 1.15–2.01), non-disclosed HIV status (OR 6.36, 95% CI: 3.58–11.29), poor drug adherence (OR 6.60, 95% CI: 1.41–30.97), poor functional status (OR 2.11, 95% CI: 1.33–3.34), being underweight (OR 2.21, 95% CI: 1.45–3.39) and advanced clinical stage (OR 1.85, 95% CI: 1.36–2.51). Whereas absence of opportunistic infections (OR 0.52, 95% CI: 0.30–0.9), normal hemoglobin status (OR 0.29, 95% CI: 0.20–0.42) and non-substance use (OR 95% CI: 0.41, 0.17–0.98) were facilitators of HIV patient retention in clinical care.

**Conclusion:**

The level of retention to the care among HIV patients was low in Ethiopia. Socio-economic, clinical, nutritional and behavioral, intervention is necessary to achieve adequate patient retention in clinical care.

## Background

Globally, 36.7 million people were living with Human Immunodeficiency Virus (HIV) in 2016 [1]. The United Nations Programme on HIV/AIDS (UNAIDS) 90–90-90 target states that 90% of people living with HIV should know their status, 90% of people living with HIV who know their status should be on treatment, and 90% of people on treatment should be virally suppressed. A record 19.5 million people were accessing antiretroviral therapy in 2017, and for the first time, more than half of all people living with HIV are on treatment [2].

In Ethiopia, Antiretroviral treatment (ART) began in 2003 and free ART was launched in January 2005. In 2016, only 67% of HIV positive people are believed to know their status. Additionally, the treatment coverage of all HIV positive people was 59% and only 51% of all HIV positive people on highly active antiretroviral therapy (HAART) had viral suppression [1]. In 2017, an estimated 738,976 Ethiopians were living with HIV and all of them were eligible for ART treatment. However, it was reported that only 426,000 were taking ARV in the same year [3].

The estimated 36-month retention from 2008 to 2013 was; 65% in Africa, 80% in Asia, and 64% in Latin America and the Caribbean [4]. In low and middle income countries HIV patient retention at 36 months on treatment averages 65–70% [4]. Patient retention in care is a challenge in many African countries [5, 6]. Similarly, in various regions of Ethiopia, during the pre-ART era, about one third of HIV positive people receiving clinical care were lost to follow up [7, 8]. Retention in care within 12 month of treatment initiation varies from 83 to 94% in Ethiopia [9].

Qualitative studies in Addis Ababa, Bahir Dar and Gondar, Ethiopia, revealed that fear of stigma, care dissatisfaction, use of holy water, fasting, and economic constraints discouraged retention in care. Whereas social support and restored health and functional ability motivated retention [10, 11].

Though Ethiopia has improved patient retention from 2005 to 2013 [12], still there are large number of patients that discontinue from treatment [13]. Unlike other regions of the world [4], there is paucity of evidence on the rate of attrition among HIV positive patient in Ethiopia. A systematic review and meta-analysis on ART treatment discontinuation of HIV patients in Ethiopia indicated various factors are responsible for treatment discontinuation [13]. However, the study was limited to only 9 papers and only three regions out of nine regions of the country. At the same time, this previous study did not include pooled incidence and prevalence of loss to follow up, death and transfer out [13]. In addition, the study does not show the magnitude of patient retention and attrition. In view of this, we decided to investigate the retention, attrition and its determinants at national level. Patient attrition includes loss to follow up (LTF), death (D) and transfer out (TO) which is the official transfer of the patient to another clinic. Therefore, this study aimed to determine the pooled magnitude of HIV patients’ clinical retention, attrition and identify factors associated with retention and attrition.

## Methods

### Study design

Systematic review and meta-analysis was done using studies conducted in English language in Ethiopia. Preferred Reporting Items for Systematic Reviews and Meta-Analyses (PRISMA) [14] was strictly followed during search of studies and analysis process.

### Eligibility criteria

We included studies conducted on adult HIV positive individuals’ retention in HIV care in Ethiopia. Studies that reported lost to follow up, defaulters and treatment discontinuation, transfer out and death were included. Studies that used any length of follow up period were eligible for this review. There was no restriction based on study designs of primary studies except systematic review and meta-analysis. Both published and unpublished primary studies from the start of ART program in January 2005 in Ethiopia to last date of literature review on June 6, 2019 were included. The reason for restriction of study period was that the first article in Ethiopia about HIV patients’ retention in clinical care data were collected in 2005 and published in 2008 and the last date of literature review search was on Jun 6, 2019. Moreover, qualitative studies which didn’t report quantitative outcomes of interest were excluded.

### Search strategy

Published research was identified through a systematic search of PubMed, Google Scholar and the African Journals Online (AJOL). Information were retrieved from online published data sources and unpublished thesis works. Online databases include PubMed, Google scholar and African Journals online (AJOL). We used search terms in PubMed as *(retention) OR attrition) AND HIV) OR AIDS) AND patients) OR positive people) AND Ethiopia).* Moreover, keywords for review included; retention, attrition, loss to follow up, dead, discontinuation, defaulters and treatment outcomes of ART in Ethiopia and were used to search additional articles. We audited the references of all articles which deemed important for our outcomes of interest. Moreover, we searched for unpublished works in the postgraduate library of Debre Markos University and other universities institutional repositories.

### Study selection and quality appraisal

Studies were evaluated using title, abstract and full text. Studies that used retrospective follow up design were included only if full cohort assessment report was obtained. Quality was assessed using Critical Appraisal and Assessment of Methodological Quality of Studies for Systematic Review and Meta-analysis of Observational Epidemiological Studies Reporting Prevalence and Cumulative Incidence Data [15]. Two authors independently assessed the quality of the studies. Where disagreement occurred, studies were discussed until consensus was reached. Studies were assessed on nine dimensions and for each dimension the authors would indicate that the criteria was met or not met. In cases where there was insufficient information to determine the study could be graded “unclear.” The following criteria were used: 1. was the sample frame appropriate to address the target population? 2. Were study participants sampled in appropriate way? 3. Was the sample size adequate? 4. Were the study subjects and the setting described in detail? 5. Was the data analysis conducted with sufficient coverage of the identified sample? 6. Were valid methods used for the identification of the condition? 7. Was the condition measured in a standard, reliable way for all participants? 8. Was there an appropriate statistical analysis? 9. Was the response rate adequate, and if not, was the low response rate managed appropriately [15]. To be included in the meta-analysis, articles were required to meet at least 5 of the nine criteria. Quality appraisal table can be found in (Additional file 1).

### Data extraction

Relevant data was extracted from eligible studies and entered into Microsoft Excel. Data collected included authors, year of publication, study period, study area, sample size, study design, our outcome of interest (retention, loss to follow up, transfer out and death), and length of follow up period.

### Measurement of outcomes

Reported rates of retention in care, loss to follow-up, death and transfer out were collected from included studies. The rate of retention in care was defined as the rate of persons in the study who remain in care across periods of follow-up which was no missed visit of clinical schedule for more than three consecutive months. Study attrition was defined as the number of patients who were lost to follow-up, transferred out, or died [16]. While LTFU was defined as HIV positive patients who miss scheduled visits to the clinic for more than three consecutive months after the last visit [17]. Death and transfer out were defined as confirmed in the patient’s record by the clinicians who were in charge of care [18]. In order to facilitate analysis, all rates were changed into100 person years of observation.

Where available, statistically significant measures of association were collected for factors associated with retention in care or attrition from care identified by studies included in the study. Then pooled odds ratio was calculated using frequency values from the primary studies and identified factors were classified as being either socio-demographic or clinical factors associated with patient retention or attrition.

### Heterogeneity and publication bias

Study heterogeneity was assessed by calculating *I*^*2*^ test statistics. Statistical significance was set at *p* < 0.05 *I*^*2*^ scores were classified into low, moderate and high inconsistency based on the I^2^ value of less than 25, 50 and 75% respectively [19]. Random effect analysis was carried out to determine the pooled estimates of patient retention and attrition. Publication bias was assessed using Egger’s test. For meta-analysis result with statistically significant publication bias, the Duval and Tweedie nonparametric trim and fill analysis using the random effect method of analysis was conducted [20].

### Statistical methods and analysis

Data analysis was conducted using Stata version 14. Pooled prevalence of LTFU, death and transfer out were estimated using each study prevalence and standard error with 95% confidence interval (CI). For LTFU, we used both pooled prevalence and incidence density separately since most of the primary studies were follow up studies. Separate meta-analysis was conducted for the LTFU, death and transfer out outcomes. The result of meta-analysis was presented using forest plots. The meta-analysis was conducted using the random effects model of analysis since it minimizes heterogeneity of the included studies [21]. Subgroup analysis was conducted for the incidence rate of loss to follow up. Studies were stratified into subgroups based on length of follow up,

## Results

A total of 3910 records were identified through online database. Of these, 1820 were identified through PubMed 900 were identified through Google Scholar, 980 were identified through Google Search and 200 from AJOL. An additional 2 articles were identified through unpublished sources. A total of 3912 were screened for inclusion. One hundred twenty were found to be duplicates and removed. Three thousands seven hundred ninety two were screened for inclusion and 3717 were excluded by title and abstract review. The full text of 75 articles were assessed for eligibility. Of these, only 45 articles passed the minimum quality score of 5 out of 9 points and were included in the meta-analysis. A quality appraisal table can be found in (Additional file 1).

### Characteristics of included studies

**Fig. 1:**
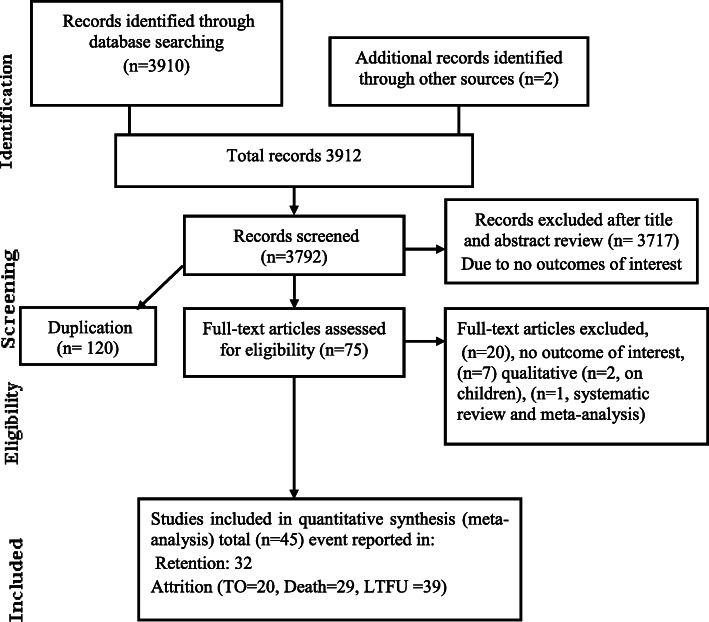
Studies included in systematic review and meta-analysis of HIV patients attrition and its determinants in Ethiopia

Most (32) of the designs of the primary articles was retrospective follow up [9, 10, 16–18, 22–48], five case control [8, 49–52], six prospective cohort study [37, 53–58] and two cross sectional survey designs [59, 60]. Every region of Ethiopia was represented in the included studies. A total of 546,250 study participants were included from 45 articles Fig. 1. Moreover, study characteristics can be found in Table 1.

**Table 1:** Characteristics of included articles (*n* = 45)

Authors and year	(n)	Study design	Study area	Major objectives of the study	Major findings
Takele et al. [24]	542	Retrospective cohort	Bichena Health center, Northwest, Ethiopia	To assess the time to lost follow up and its predictors among adult HIV positive people receiving ART	Prevalence of lost to follow up was 40.8% and incidence was 13.45/100 person years. Predictors of loss to follow up were: ➢ Poor drug adherence AHR, 2.91 (2.08–4.09), ➢ TDF based base line regimen AHR, 1.63 (1.20–2.20), ➢ baseline regimen change AHR, 1.79 (1.08–2.97) ➢ Poor functional status AHR, 2.71 (2.01–3.66)
Yigzaw et al. [22]	484	Retrospective cohort	Debre Markos referral hospital, Northwest, Ethiopia	To determine the incidence and predictors of loss to follow up among HIV positive adults on ART	About 17.36% of the individual were lost from ART follow-up. Rate of lost to follow-up was found to be 3.7 per 100 person- year’s observation and 30.95% occurred within the last years of follow up. The predictors for LTFU were: ➢ ART Regimen **[(**AZT-3TC-NVP, AHR (95% CI = 2.79 (1.07, 7.23)), ➢ AZT-3TC-EFV, AHR (95% CI) = 3.14 (1.13, 8.08) ➢ TDF-3TC-EFV, AHR (95%CI = 9.3 (3.75, 23.8)], ➢ good ART adherence [AHR (95%CI) = 0.54 (0.3, 0.9)], ➢ WHO clinical stage IV [AHR: 95%CI = 2.75 (1.23–6.16)], ➢ Urban residence [AHR: 95%CI) = 0.6 (0.37–0.99), ➢ no cell phone [AHR: 95%CI) = 1.9 (1.14–3.4)] ➢ Age categories [(35–44 years (AHR(95%CI) = 0.32 (0.15–0.67**)** ➢ 45+ years (AHR (95%CI) =0.33 (0.13–0.83)].
Seifu et al. [16]	1439	Retrospective cohort	Karamara general hospital, Jigjiga town, Eastern Ethiopia	To measure incidence and predictors of loss to follow up among adult ART clients.	The incidence rate of loss to follow up in the cohort was 26.6% (95% CI; 18.1–29.6) per 100 person months. Factors associated with LTFU were; ➢ Patients with male sex [HR: 2.1CI;(1.3–3.4)] ➢ patients whose next appointment weren’t recorded [HR: 1.2, 95% CI; (1.12–1.36)] ➢ patients who did not disclose their status to any one [HR: 2.8, 95% CI; (2.22–5.23)]
Berheto et al. [23]	8009	Retrospective cohort	Four health centers and 2 hospitals in Southern, Ethiopia	To examine the effects of mental health training on HIV patient retention in care.	The incidence of attrition was 6.5 per 100 person-years ➢ 21% higher in the unexposed group (HR 1.21; 95% CI 1.1, 1.3) ➢ Retention in care was significantly higher in the mental health trained group. Independent risk factors for attrition were; ➢ WHO clinical staging III/IV ➢ Tuberculosis co-infection ➢ the male gender ➢ Poor functional status
Assemie et al. [17]	602	Retrospective cohort	Pawi General Hospital, northwest Ethiopia	To assess incidence of lost-to-follow-up and its predictors among HIV-positive adults after initiation of ART	Cumulative incidence of lost-to-follow-up after ART initiation was high, 11.6 (95% CI 9.8–13.7) per 100 adult-years follow-up time. Independent significant predictors of lost to follow up were; ➢ Being aged 15–28 years (AHR = 0.44; 95% CI 0.24–0.83) ➢ being on WHO clinical stage IV (AHR = 2.09; 95% CI 1.02–3.13) ➢ Receiving isoniazid preventive therapy (AHR = 0.11; 95% CI 0.06–0.18).
Adewo et al. [25]	652	Retrospective cohort	Tepi General Hospital in South West Ethiopia.	to assess factors related with time to attrition	179 patients were lost to follow up and 37 patients died, contributing to an overall attrition of 33.13%. During the early six months the attrition rate was 89.8%. ➢ Not starting cotrimoxazole prophylaxis (AHR = 1.51, 95% CI, 1.02–2.25) ➢ being co-infected with tuberculosis (TB) (AHR = 2.16, 95%CI, 1.35–3.45) ➢ living further than 10 km away from the hospital (AHR = 1.44, 95%CI, 1.07–2.0) ➢ Not disclosed status of HIV (AHR = 3.04) were factors significantly associated with time to attrition.
Gesesew et al. [26]	3607	Retrospective cohort	Jimma University Teaching Hospital, Western Ethiopia	to assess prevalence, trend and risk factors for ART discontinuation	1090 (22.3%) had discontinued, 954 (19.5%) had transferred out, 300 (6.1%) had died, 2517 (51.4%) were alive and on ART. The trend of ART discontinuation showed an upward direction in the recent times and reached a peak, accounting for a magnitude of 10%, in 2004 and 2005. ➢ Being a female (AOR = 2.1, 95%CI: 1.7–2.8), ➢ Having an immunological failure (AOR = 2.3, 1.9–8.2) ➢ having tuberculosis/HIV co-infection (AOR = 1.5, 1.1–2.1) ➢ No previous history of HIV testing (AOR = 1.8, 1.4–2.9) were the risk factors for ART discontinuation.
Bucciardini et al. [53]	1198	prospective cohort	In seven health facilities in Tigray in northern Ethiopia.	To determine predictors of patient attrition after 12 months in care	Kaplan–Meier estimates of retention in care were 83.9, 82.1 and 79.8% at 12, 18 and 24 months after starting ART, respectively. Attrition was mainly due to loss to follow-up (6.8%), transferred-out patients (9.5%) and mortality (4.4%). Factors associated with attrition were; ➢ Male sex ➢ CD4 count < 200 cells/μL ➢ Type of health facility
Wilhelmson et al. [27]	383	retrospective cohort	Adama Hospital, central Ethiopia	To determine retention in care among patients receiving second-line ART in a public hospital	At the end of study follow-up, 80.5% of patients remained in care (adults and adolescents 79.8%; children 85.7%). LTFU among adults and adolescents was associated with; ➢ baseline CD4 cell count 100 cells/mm3 and ➢ First-line regimen failure that was not confirmed by HIV RNA testing.
Tiruneh et al. [10]	222	retrospective cohort	Addis Ababa, Central Ethiopia	To assessed how well patients stay in care and explored factors associated with retention	Thirty percent were LTFU by end of the study; the median time to LTFU was 1675 days. Higher risk of LTFU was associated with: ➢ Baseline CD4 counts < 100 and > 200 cells/uL (HR = 1.62; 95%CI: 1.03–2.55; and HR = 2.06; 95%CI: 1.15–3.70, compared with patients with baselineCD4 counts of 100–200 cells/uL. ➢ Bedridden at baseline (HR = 2.05; 95%CIs [1.11–3.80]) ➢ Those with no or only primary education (HR = 1.50; 95%CIs [1.00–2.24]) were more likely to be LTFU. ➢ The qualitative data revealed that fear of stigma, care dissatisfaction, use of holy water, and economic constraints discouraged retention in care.
Megerso et al. [49]	1248	Case control	Oromia, central and western Ethiopia	identifying correlates of loss to follow-up in ART among adult patients	factors which increased the risk of loss to follow-up in ART were; ➢ Age 15–24 years [AOR], 19.82 95% CI: 6.80, 57.73); ➢ day laborers (AOR, 5.36; 95% [CI]: 3.23, 8.89), ➢ rural residents (AOR, 2.35; 95% CI: 1.45, 3.89), ➢ WHO clinical stage IV (AOR, 2.29; 95% CI: 1.45, 3.62), ➢ Baseline CD4,350 cells/mL (AOR, 2.06; 95% CI: 1.36, 3.13), ➢ suboptimal adherence of ART (AOR, 7.42; 95% CI: 1.87, 29.41)
Mitiku et al. [28]	346	retrospective cohort	South and North Wollo, Oromia special zone, Northern Ethiopia	To determine levels and determinants of LFU under Option B+ among pregnant and breastfeeding women	Overall, 57 (16.5%) women were LTFU. The cumulative proportions of LTFU at 6, 12 and 24 months were 11.9, 15.7 and 22.6%, respectively. ➢ The risk of LTFU was higher in younger women 18 to 24 years than 30 to 40 years: (AHR = 2.3; 95% (CI): 1.2 to 4.5) ➢ In those attending hospitals compared to those attending health centers (AHR: 1.8; 95% CI: 1.1 to 3.2), ➢ In patients starting ART on the same day of diagnosis (AHR: 1.85; 95% CI: 1.1 to 3.2) ➢ Missing CD4 cell counts at ART initiation (AHR: 2.3; 95% CI: 1.2 to 4.4).
Teshome et al. [54]	1173	retrospective cohort	22 hospitals and 25 health centers in southern Ethiopia.	compared death and loss to follow-up (LTFU) rates among ART patients among patients in hospitals and health centers	24.6% were either dead or LTFU, resulting in a retention rate of 75.4%. The death rates were 3.0 and 1.5 and the LTFU rate were 9.0 and 10.9 per 100 person-years of observation in health centers and hospitals, respectively. The competing-risk regression model showed that; ➢ The longer gap between testing and initiation of ART, ➢ body mass index > 18.5 (AHR, 0.58 (95% CI, 0.38–0.91) ➢ advanced WHO clinical stage ➢ No Isoniazid prophylaxis (AHR, 1.90 (95% CI, 1.10–3.23) ➢ Age 26–39 (AHR, 0.59 (95% CI, 0.42–0.83) ➢ Secondary and above educational compared to no education status (AHR, 0.58 (95% CI, 0.39–0.67) were independently associated with LTFU. ➢ Moreover, baseline tuberculosis disease, poor functional status /bed ridden (AHR, 5.35 (95% CI, 1.67–17.1), and follow-up at a health center were associated with an elevated probability of death.
Dessalegn et al. [50]	727	Case control	Wukro primary public hospital, Northern Ethiopia	to assess the magnitude and predictors of loss to follow-up among adult ART clients	11% of them were loss to follow up. Factors associated with LTF were: ➢ Absence of bereavement concern AOR, 0.12 (0.046,0.30) ➢ not provided Isoniazide (INH) prophylaxis AOR, 3.04 (1.3, 7.3), ➢ The presence of side effects AOR, 12.34 (4.86, 31.35) ➢ Earlier (< 36 month) periods after ART AOR, 23.54 (8.87, 62.45)
Melaku et al. [30]	93,418	retrospective cohort	Nation wide	To measure trend and treatment outcomes of HIV treatment	24% of patients were LTF before ART initiation. Among those initiating ART, attrition was 30% after 36 months, with most occurring within the first 6 months. Recorded death after ART initiation was 6.4 and 9.2% at 6 and 36 months, respectively, and decreased over time. Younger age, male gender, never being married, no formal education, low CD4+ cell count, and advanced WHO stage were associated with increased LTFU. Death was lower among younger adults, females, married individuals, those with higher CD4+ cell counts and lower WHO stage at ART initiation
Shaweno et al. [29]	626	retrospective cohort	Sheka Zonal Hospital, southern Ehtiopia	time when LTFU occurs and the associated factors among adults enrolled in pre-ART care	A total of 178 (28.4%) pre-ART patients were lost to Follow up, 93% of which occurred within the first six months. The independent predictors included: ➢ Not having been started on cotrimoxazole prophylaxis [AHR] = 1.77, 95%, [CI], 1.12–2.79), ➢ baseline CD4 count of or above 350 cells/mm3 (AHR = 1.87, 95%CI, 1.02–3.45) ➢ An undisclosed HIV status (AHR = 3.04, 95%CI, 2.07–4.45).
Mekuria et al. [31]	836	retrospective cohort	Addis Ababa, Central Ethiopia	To describe the proportion of patients who are retained in HIV care and characterize predictors of attrition among HIV-infected adults receiving cART	Nearly 80% (95%CI: 76.7, 82.1) of the patients were retained in care in the first 3 and half years of antiretroviral therapy. After successfully tracing more than half of the LTFU patients, the updated one year retention in care estimate became 86% (95% CI: 83.41, 88.17%). ➢ Severe immune deficiency at enrolment in care/or at ART initiation ➢ ‘bed-ridden’ or ‘ambulatory’ functional status at the start of ART predicted attrition.
Bucciardini et al. [55]	563	Prospective cohort	Tigray, Northern Ethiopia	We report data on retention in care and its associated determinants	Overall 85.1% of their patients retained after one year from starting ART. Loss to follow-up (5.5%) and transfers to other health facilities (6.6) were the main determinant of attrition. The factors associated with retention were; ➢ The type of health facilities, ➢ Active TB (HR 1.72, 95% CI: 1.23–2.41) ➢ Male gender (AHR, 1.34 (955 CI, 1.04 to 1.7)4
Assefa et al. [32]	11,371	retrospective cohort	three health facilities in Addis Ababa, Central Ethiopia	To identify the level of long-term outcomes and their determinants in patients on ART in Ethiopia	Retention rates were 82, 74, and 72% at 24, 60, and 84 months on ART, respectively. Retention was associated with: ➢ Male sex, adolescent age, marital status, advanced HIV disease, Illiteracy and peer-support services ➢ However, long-term retention was associated independently with: ➢ only male sex (AHR) 0.68 (0.56 to 0.77)] ➢ married patients [with AHR 0.62 (0.54 to 0.72)] ➢ peer-support services [with AHR 1.62 (1.58 to 1.66)]
Berheto et al. [61]	2133	retrospective cohort	Mizan-Aman General Hospital in the Southern Ethiopia	aimed at determining the incidence and risk factors for LTFU in HIV patients on ART	Around 574 (26.7%) patients were defined as LTFU. The cumulative incidence of LTFU was 8.8 (95% CIs 8.1–9.6) per 1000 person months. ➢ Patients with regimen substitution (HR 5.2; 95% CIs 3.6–7.3), ➢ Never took isoniazid (INH) prophylaxis (HR 3.7; 95% CIs 2.3–6.2), ➢ adolescent (HR 2.1; 95% CIs 1.3–3.4), ➢ Had a baseline CD4 count < 200 cells/mm3 (HR 1.7, 95% CIs 1.3–2.2) were at higher risk of LTFU, WHO clinical stage III (HR 0.6; 95% CIs 0.4–0.9) and IV (HR 0.8; 95% CIs 0.6–1.0) patients at entry were less likely to be LTFU than clinical stage I patients ➢ Ambulatory and bedridden functional status were less affected by LTF than working groups ((HR, 0.4 (0.3,0.6) and HR, 0.7 (0.5, 0.9)) respectively ➢ There was no significant difference in risk of LTFU in males and females in this study
Reepalu et al. [34]	678	Prospective cohort	Five health centers in Adama, Central Ethiopia	compared virological suppression (VS) rates, mortality, and retention in care in HIV-positive adults receiving care	No difference in retention in care between TB and non-TB patients was observed during follow-up; 25 (3.7%) patients died, and 17 (2.5%) were lost to follow-up (*P* = .30 and *P* = .83, respectively).
Tadesse et al. [33]	520	retrospective cohort	Tigray, Northern Ethiopia	To determine loss to follow up and its determinants	51 (9.8%) were loss giving a LTFU rate of 8.2 per 100 person- years. From these LTFU, 21 (41%) occurred within the first Six months of ART initiation. The independent predictors of LTFU of patient were: ➢ being smear positive pulmonary TB [AHR (95% CI) = (2.05 (1.02, 4.12)], ➢ male gender [AHR (95%CI) = (2.73 (1.31, 5.66)], ➢ regiment AZT-3TC-NVP [AHR (95%CI) = (3.47 (1.02,11.83)] and ➢ Weight ≥ 60 kg [AHR (95% CI) = (3.47 (1.02, 11.83)].
Wubshet et al. [59]	3012	Cross sectional	Gondar, Northwest Ethiopia	To determine the outcome and factors associated with LTF among HIV patients	Out of the 551 patients LTF, 486 (88.20%) were successfully tracked. Death was the most common reason accounted for 233 (47.94%) of the lost to follow-up. Reasons for non-deaths losses include: stopped antiretroviral treatment due to different reasons, 135 (53.36%), and relocation to another antiretroviral treatment program by self- transfer, 118 (46.64%). The rate of mortality in the first six months was 72.12 per 100 person-years (95% CI: 61.80–84.24) but this sharply decreased after 12 months to 7.92 per 100 person-years (95% CI: 4.44–14.41). Baseline clinical characteristics were strongly associated with outcome such as presence of tuberculosis infection at ART initiation, functional status (both ambulatory and bed ridden); CD4 cell count, 100 cells/mL, and WHO stage III and IV were strongly associated with mortality. On the other hand, male sex, bedridden functional status and residence outside Gondar town were significantly associated with non-death losses.
Asefa et al. [51]	236	Case control	Nekemte Hospital, western Ethiopia	to assess determinants of defaulting from antiretroviral treatment	After controlling for possible confounders, ➢ living far from the facility (out of the town) (AOR = 4.1; 95%CI 1.86 to 9.42), ➢ dependent patients for source of food [AOR = 13.9; 95%CI 4.23 to 45.99], ➢ patients with mental status not at ease [AOR = 4.7; 95%CI 1.65 to 13.35], ➢ patients whose partners were HIV negative [AOR = 5.1; 95%CI 1.59 to 16.63], ➢ patients whose partners HIV status were unknown or not tested [AOR = 2.8; 95%CI 1.23 to 6.50] ➢ Patients that fear stigma [AOR = 8.3; 95%CI 2.88 to 23.83] were statistically significant association.
Ahmed et al. [8]	1817	Case control	Gondar, Northwest Ethiopia	To investigate factors associated with pre-ART LTFU in Ethiopia.	factors were found to be independently associated with pre-ART LTFU: ➢ male gender (AOR) = 2.00 (95% CI: 1.15, 3.46)] ➢ higher baseline CD4 cell count (251–300 cells/μl [AOR = 2.64 (95% CI: 1.05, 6.65)]; 301–350 cells/μl [AOR = 5.21 (95% CI: 1.94, 13.99)], and > 350 cells/μl [AOR = 12.10 (95% CI: 6.33, 23.12)] compared to CD4 cell count of ≤200 cells/μl) ➢ Less advanced disease stage (WHO stage I [AOR = 2.81 (95% CI: 1.15, 6.91)] compared to WHO stage IV). ➢ Married patients [AOR = 0.39 (95% CI: 0.19, 0.79)] had reduced odds of being LTFU. ➢ Patients whose next visit date was not documented on their medical chart [AOR = 241.39 (95% CI: 119.90, 485.97)]
Wubshet et al. [60]	3012	Survey	Gondar, Northwest Ethiopia	to evaluate mortality, loss to follow up, and retention in care	61.4% of the patients were retained on treatment, 10.4% died, and 31.4% were lost to follow up. Fifty-six percent of the deaths and 46% of those lost to follow up occurred in the first year of treatment. ➢ Male gender (AHR) 3.26; 95% CI: 2.19–4.88) ➢ CD4 count≤200 cells/μL (AHR 5.02; 95% CI: 2.03–12.39), ➢ tuberculosis (AHR 2.91; 95% CI: 2.11–4.02); ➢ bed-ridden functional status (AHR 12.88; 95% CI: 8.19–20.26) were predictors of mortality, ➢ Whereas only CD4 count < 200 cells/μL (HR = 1.33; 95% CI: (0.95, 1.88) and ambulatory functional status (HR = 1.65; 95% CI: (1.22, 2.23) were significantly associated with LTF.
Assefa et al. [36]	37,466	retrospective cohort	Nationwide study	Intended to evaluate the outcomes of the ART services in 55 health facilities in Ethiopia.	Health facilities were able to retain 29,893 (80%), 20,079 (74%) and 5069 (68%) of their patients after 6, 12 and 24 months on ART, respectively. Retention rates vary across health facilities, ranging from 51 to 85% after 24 months on ART. Mortality was 5, 6 and 8% after 6, 12 and 24 months on ART. More than 79% of patients with available CD4-cell counts had a baseline CD4-cell counts less than 200 cells per micro-liter of blood.
Balcha et al. [37]	1709	Prospective cohort	Oromia region	to compare the outcomes of antiretroviral therapy (ART) between hospital and health center levels	1044 (61%) remained alive and were on treatment after 24-month follow-up. In all, 835 (57%) of ART patients at hospitals and 209 (83%) at health centers were retained in the program. Of those who were alive and receiving ART, 79% of patients at health centers and 72% at hospitals were clinically or immunologically improving. In addition, 331 (23%) patients at hospitals were LFTU as compared to 24 (10%) of patients at health centers (relative risk [RR] at 95% confidence interval [CI]: .358 [.231–.555]). While 11% was the mortality rate at hospitals, 5% of patients at health centers also died (RR at 95% CI: .360 [.192–.673]).
Deribe et al. [52]	1270	Case control	Jimma University Teaching Hospital, western Ethiopia	To determine the prevalence of and factors associated with defaulting from antiretroviral treatment (ART)	Of 1270 patients who started ART, 915 (72.0%) were active ART users and 355 (28.0%) had missed two or more clinical appointments. The latter comprised 173 (13.6%) defaulters, 101 (8.0%) who transferred out, 75 (5.9%) who died, and 6 (0.5%) who restarted ART. Reasons for defaulting were unclear in most cases. Reasons given were; loss of hope in medication, lack of food, Mental illness, holy water, no money for transport and other illnesses. Tracing was not successful because of incorrect address on the register in 61.6% of the cases. ➢ Taking hard drugs (cocaine, cannabis and IV drugs) ➢ excessive alcohol consumption ➢ Being bedridden, ➢ living outside Jimma town ➢ Having an HIV negative or unknown HIV status partner were associated with defaulting ART.
Abebe et al., [18]	640	Retrospective cohort	Debre Markos referral hospital, Northwest Ethiopia	To determine Survival status of HIV positive adults on antiretroviral treatment	640 patient cards (379 alive and 261 death) adult HIV infected individuals were included in the study. General mean estimated survival time of patients after HAART initiation was improved. Significant predictors of mortality after HAART initiation were: Lower baseline hemoglobin; Ambulatory and bed ridden functional status; Poor ART adherence; Advanced WHO clinical stage; Absence of recent TB prophylaxis; Unrecognized side effects; Persistent unexplained chronic diarrhea (> 1 month)
Ahmed et al. [47]	503	Retrospective cohort	Afar, north-east Ethiopia.	assessed the incidence of tuberculosis (TB) and its predictors among adults living with HIV/AIDS	40 transfer out to other health facilities 13 died 21 loss to follow up 258 did not develop TB 119 developed TB
Mekonnen et al. [46]	1533	Retrospective cohort	Jimma hospital southwest Ethiopia	to assess reasons and predictors of regimen change from initial highly active antiretroviral therapy	One in two (47.7%) adults changed their antiretroviral therapy regimen. Patients who were above the primary level of education [Hazard ratio (HR) 1.241 (95% CI 1.070–1.440)] and with human immunodeficiency virus/Tuberculosis co-infection [HR 1.405 (95% CI 1.156–1.708)] had the higher risk of regimen change than their comparator.
Chaka et al. [40]	248	Retrospective cohort	Adama, Central Ethiopia	to assess Option B+ PMTCT service intervention outcomes.	Loss to follow-up from the Option B+ continuum was 10 (4.2%).
Tadege [41]	1512	Retrospective cohort	Mettu Karl Hospital, southwest Ethiopia	to determine the major risk factors of antiretroviral therapy dropout	the risk of dropout for patients with primary education status was 10.58% greater as compared to illiterate (*p* < 0.0110). The probability of dropout for patients with marital status separated was about 16.82% higher than those patients with marital status divorced (*p* < 0.0070). Being merchant, farmer and daily labour had a greater risk of dropout as compared to a housewife.
Gezea et al. [39]	305	Retrospective cohort	Mekelle, Northern Ethiopia	aimed at investigating the incidence and predictors of LTFU of TB/HIV co-infected patients	45 of 305 (14.8%) of TB/HIV co-infected adults were LTFU with an incidence rate of 4.5 new LTFUs per 100 Person Years (PYs) and a median follow up time of 3.1 years (Interquartile Range (IQR): 0.8–5.3 Years). Hemoglobin level ≤ 11.0 g/dl (AHR = 2.660; 95%CI: 1.459–4.848), and any history of OI/s (AHR = 3.795; 95%CI: 1.165–12.364) were risk factors of LTFU. While, adverse drug events (AHR = 0.451; 95%CI: 0.216–0.941), TB treatment completion (AHR =0.121; 95% CI: 0.057–0.254), and being on Isoniazid Preventive Therapy (IPT) (AHR = 0.085; 95%CI: 0.012–0.628) had protective effect against LTFU.
Mekonnen et al. [38]	569	Retrospective cohort	Gondar, Northwest Ethiopia	to estimate the incidence of lost to follow up from ART care and identify the associated factors among HIV infected patients after first-line ART initiation	The overall incidence rate of lost to follow up was 12.26 per 100 person years (95% CI (10.61–14.18)). Being underweight (< 18.5 kg/m2) (AHR, 1.52, 95% CI 1.01–2.28), jobless (AHR, 2.22, 95% CI 1.2–4.11), substance abuser (AHR, 1.84 95% CI 1.19–2.86), having sub-optimal adherence (fair/poor) (AHR 6.33, 95% CI (3.90–10.26)), not receiving isoniazid prophylaxis (AHR 2.47, 95% CI (1.36–4.48)), ambulatory functional status (AHR 1.94, 95% CI (1.23–3.06)), having opportunistic infections (AHR, 1.74 95% CI 1.11–2.72), having CD4 count 201–349 cells/μL (AHR 0.58, 95% CI (0.38–0.88)) were found to be significant predictors of lost to follow up from ART service.
Damitew et al. [44]	784	Retrospective cohort	Kharamara hospital, Somalia, Eastern Ethiopia	to assess survival and identify predictors of death in adult HIV-infected patients initiating ART	There were 87 (11.1%) deaths yielding an overall mortality rate of 5.15/100 PYO (95% CI: 4.73–6.37). The estimated mortality was 8.4, 9.8, 11.3, 12.7 and 14.1% at 6, 12, 24, 36 and 48 months respectively. The independent predictors of death were single marital status (AHR: 2.31; 95%CI: 1.18–4.50), a bedridden functional status (AHR: 5.91; 95%CI: 2.87–12.16), advanced WHO stage (AHR: 7.36; 95%CI: 3.17–17.12), BMI < 18.5 Kg/m2 (AHR: 2.20; 95%CI: 1.18–4.09), CD4 count < 50 cells/μL (AHR: 2.70; 95%CI: 1.26–5.80), severe anemia (AHR: 4.57; 95%CI: 2.30–9.10), and TB co-infection (AHR: 2.30; 95%CI: 1.28–4.11)
Ayele et al. [43]	730	Retrospective cohort	Kembata and Hadiya zones	To assesses treatment outcomes and its determinants for HIV patients on ART	A total of 92 (12.6%) patients died, 106 (14.5%) were lost to follow-up, and 109 (15%) were transferred out. Sixty three (68%) deaths occurred in the first 6 months of treatment. The median survival time was 25 months with IQR [9, 43]. After adjustment for confounders, WHO clinical stage IV [HR 2.42; 95% CI, 1.19, 5.86], baseline CD4 lymphocyte counts of 201 cell/mm3 and 350 cell/mm3 [HR 0.20; 95% CI; 0.09–0.43], poor regimen adherence [HR 2.70 95% CI: 1.4096, 5.20], baseline hemoglobin level of 10 g/dl and above [HR 0.23; 95% CI: 0.14, 0.37] and baseline functional status of bedridden [HR 3.40; 95% CI: 1.61, 7.21] were associated with five year survival of HIV patients on ART.
Bezabh et al. [57]	337	Retrospective cohort	Bahrdar and Gondar, northwest Ethiopia	to determine patient, regimen, disease, patient-provider, and healthcare-related factors associated with adherence with ART	130 (75.6%) had ≥95% adherence. In the multivariate analyses, a higher baseline BMI (OR, 1.2; 95% CI 1.0, 1.4) and use of reminder devices (OR, 9.1; 95% CI 2.0, 41.6) remained positively associated with adherence
Awoke et al. [42]	2386	Retrospective cohort	Gondar, northwest Ethiopia	to determine and compare the long-term response of patients on nevirapine and efavirenz	70.58% were retain in clinical care 302 were transfer out to other health facilities 230 were lost to follow up 170 were dead
Mekuria et al. [48]	870	Retrospective cohort	Addis Ababa, Central Ethiopia	To investigated virological suppression levels and its predictors of detectable viraemia	A total of 656 (75.4%) patients, who were alive, were retained in HIV care. Virological suppression levels can be high in an established ART programme in a resource-limited setting
Assefa et al. [9]	20,099	Retrospective cohort	Nation wide	To reviews the performance of the ART program in Ethiopia	The ART program has been successful over several critical areas: (1) ART coverage improved from 4 to 54%; (2) the median CD4 count/mm3 at the time of ART initiation increased from 125 in 2005/ 6 to 231 in 2012/13; (3) retention in care after 12 months on ART has increased from 82 to 92%. In spite of these successes, important challenges also remain: (1) ART coverage is not equitable: among regions (5.6–93%), between children (25%) and adults (60%), and between female (54%) and male patients (69%); (2) retention in care is variable among regions (83–94%); and, (3) the shift to second-line ART is slow and low (0.58%).
Lifson et al. [56]	142	Prospective cohort	Arbaminch	The effects of community based support on patient retention	Community health support workers (CHSWs) provided HIV and health education, counseling/social support, and facilitated communication with the HIV clinics. With 7 deaths and 3 transfers, the 12-month retention rate was 94% (95% CI ¼ 89–97%), and no client was LTFU in the project. Between enrollment and 12 months, clients had significant (*P* < .001) improvements in HIV knowledge (17% increase), physical and mental quality of life (81 and 21% increase), internalized stigma (97% decrease), and perceived social support (24% increase).
Tadege [45]	600	Retrospective cohort	Illubabur	Time to death predictors	The risk of death for patients who lived with tuberculosis was about 2.872-fold times higher than those patients who were negative. Most of the HIV/AIDS patients on antiretroviral therapy were died in a short period due to tuberculosis comorbidity, began with lower amount of CD4, being underweight, merchant, and being on WHO clinical stage IV
Telele et al. [58]	874	Prospective cohort	Nation wide	To predict first-line ART outcome after 6 and 12 months	The treatment failure rates were 23.3 and 33.9% at 6 and 12 months, respectively. The odds of LTFU at month 6 increased with baseline functional disabilities, WHO stage III/IV, and CD4 cells < 50/μl. At month 6, 131/874 (15.0%) patients were dead (*n* = 62) or LTFU due to other reasons (*n* = 69).
Assesfa et al. [12]	334,819	Retrospective cohort	Nation wide	We aimed to analyze the ART program in Ethiopia.	While ART was being scaled up, retention was recognized to be insufficient. To improve retention, a second wave of interventions, related to programmatic, structural, socio-cultural, and patient information systems, have been implemented. Retention rate increased from 77% in 2004/5 to 92% in 2012/13.
**Total number of participants 546,250**					

Key: *AHR* Adjusted Hazard Ratio, *LTFU* Loss to follow up

### Magnitude of retention in HIV care

**Fig. 2:**
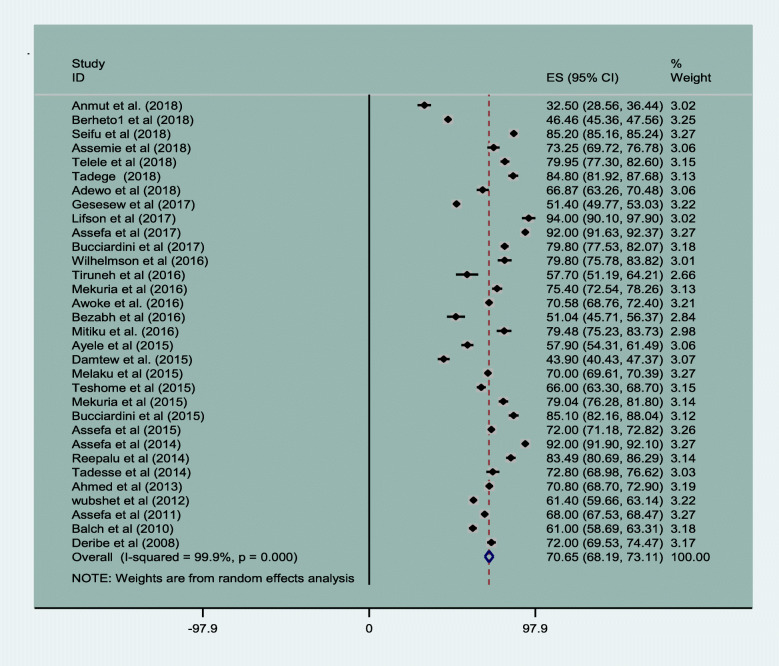
Magnitude of HIV patients retention of care in Ethiopia from 2005 to 2019

Of the 45 original articles, 32 articles reported magnitude of patient retention in HIV care in Ethiopia [8–10, 17, 23–28, 30–32, 34, 36, 37, 41–45, 48, 52–58, 60, 62, 63]. The magnitude of retention in HIV care was 70.65% (95% CI, 68.19, 73.11) with I^2^ value of 99.9% at *P*-value < 0.001. The minimum retention was 32.50% (95% CI, 28.56 to 36.44) in a 9 years follow up period [24] and the maximum retention was 94% (95% CI, 90.1 to 97.7) in 1 year. The result is given in Fig. 2.

**Fig. 3:**
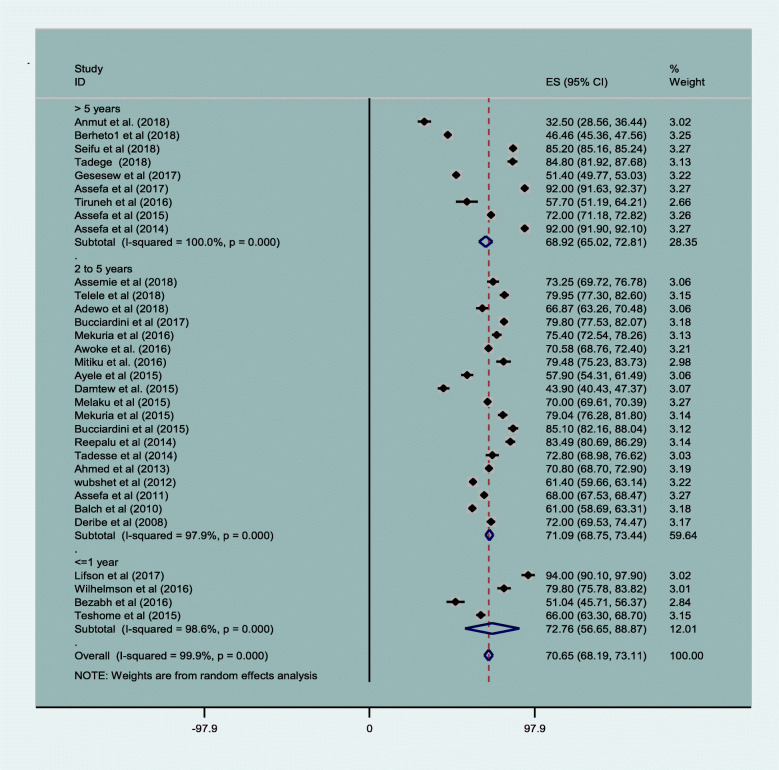
Magnitude of HIV patients’ retention by years of follow up in Ethiopia from 2005 to 2019

The maximum and minimum follow up period was 14 years and 3 months respectively. Except one article that used 3 months of follow up [27], all original studies used a follow up period of above 1 year. Most (19) of the studies that reported a follow up period from 2 to 5 years had average retention in care of 71.09% (95% CI, 68.75,73.44). Furthermore, 9 articles that used more than 5 years follow up period that reported on average retention rate of 68.92% (95% CI, 65.02, and 72.81) Fig. 3.

**Fig. 4:**
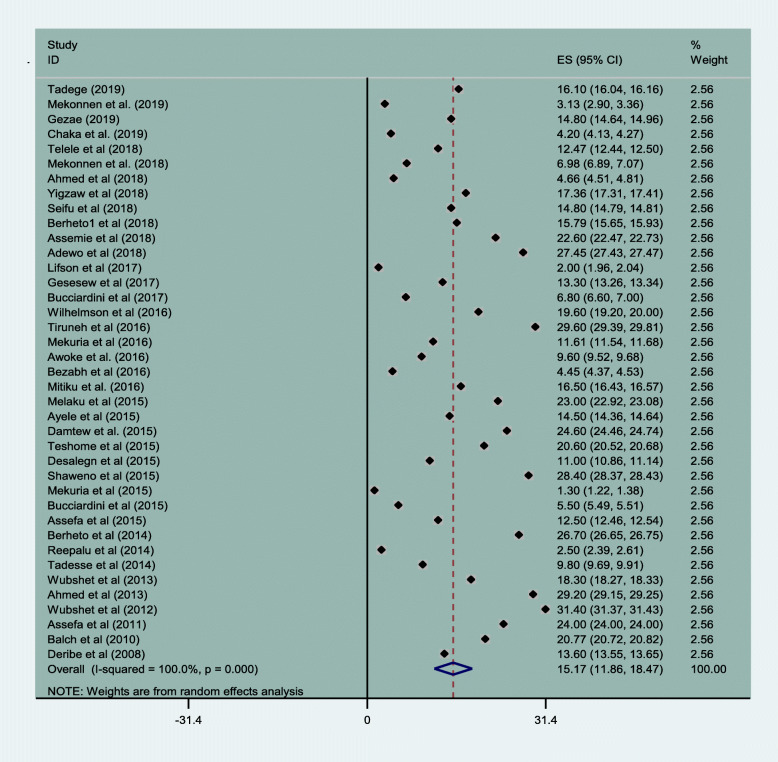
Magnitude of loss to follow up of HIV patients in Ethiopia from 2005 to 2019

The magnitude of attrition was an aggregations of LTFU, transfer out and death. Accordingly, the magnitude of loss to follow up was 15.17% (95% CI: 11.86, 18.47). The lowest and highest rate of loss to follow up was 3.13% (95% CI: 2.90, 3.36) and 31.40% (95% CI: 31.37, 31.43) Fig. 4.

### Magnitude of attrition rate of HIV/AIDS patients in Ethiopia

**Fig. 5:**
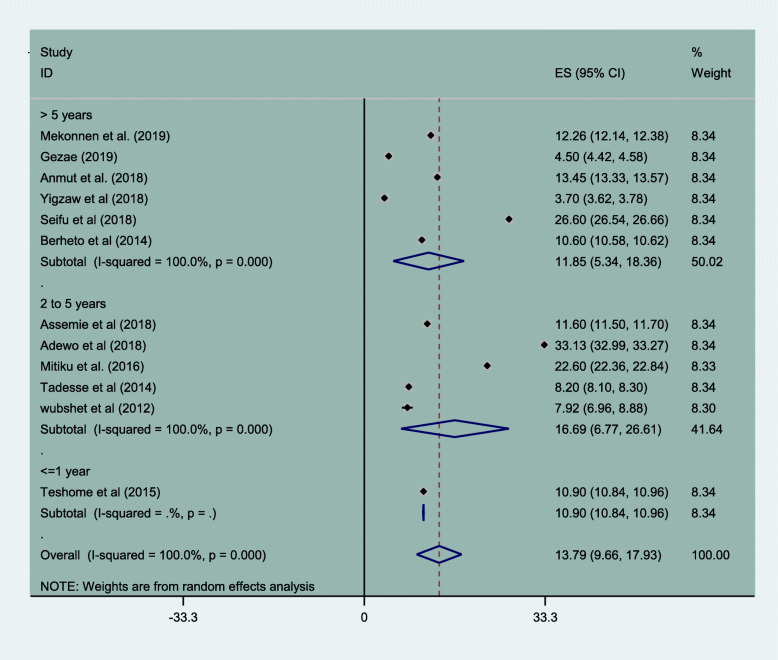
Incidence rate of loss to follow up among HIV patient on treatment in Ethiopia

Incidence of loss to follow up was 13.79/100 person years of observation (95% CI, 9.66–17.93). The incidence rate was highest in the follow up years 2 through 5 years which was 16.69/100 person years of observation, (95% CI, 6.77–26.61). Subgroup analysis of incidence rate of loss to follow up was reported by 12 papers with resulted cumulative incidence density of 13.79/100 person years of observation (95% CI, 9.66–17.93). It is higher in the follow up years ranged from 2 to 5 years 16.69/100 person years of observation, (95% CI, 6.77–26.61) Fig. 5.

Most common contributing factors for patient attrition was loss to follow up (15.17%), transfer out (12%) and death (5.71%) respectively Table 2.

**Table 2:** Summery of retention and attrition rates among HIV patients attending clinical care in Ethiopia from 2005 to 2019

Attrition rate	No. of studies	Sample	Magnitude at 95% CI
Retention rate	32	492,356	70.65 (68.19,73.11)
Magnitude of Loss to follow up	39	147,896	15.17 (11.86,18.47)
Incidence of Loss to follow up	12	11,777	13.79/100 person years of observation (9.66,17.93)
Death	29	105,699	6.75 (6.22,7.27)
Transfer out	20	34,565	11.17 (7.12,15.21)

### Mortality of HIV patients in Ethiopia

**Fig. 6:**
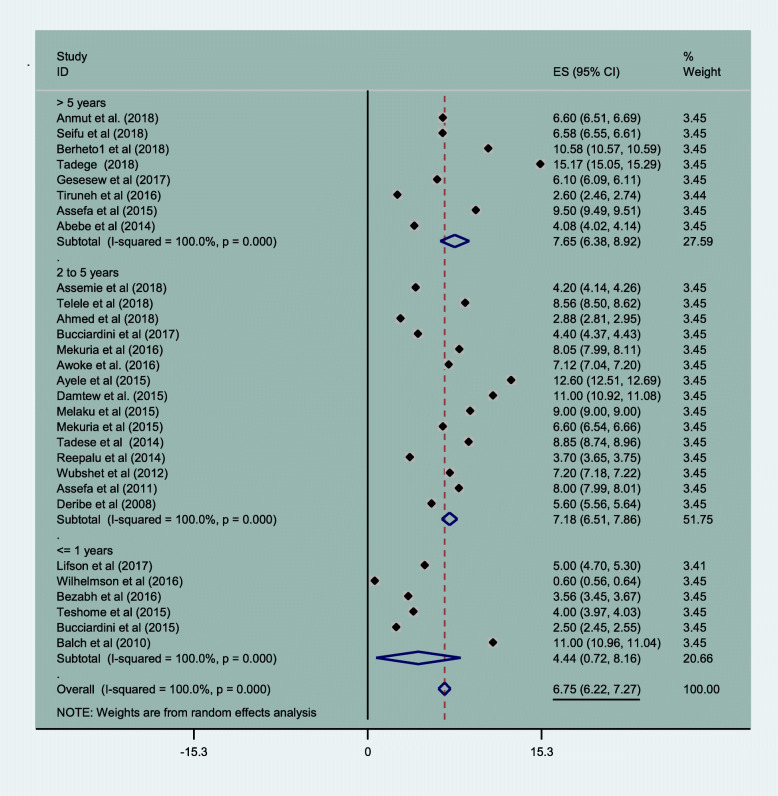
Magnitude of HIV patient mortality in Ethiopia from 2005 to 2019

Overall mortality of HIV patients in this meta-analysis was 6.75% (95% CI: 6.22, 7.27). Mortality rates were lowest in the first years of follow up (4.4% (95% CI: 0.72, 8.16)) and highest when follow up exceeded 5 years (7.65% (95% CI: 6.38, 8.92)). Pooled mortality rates are shown in Fig. 6.

### HIV patients transfer out from one to other health facility

**Fig. 7:**
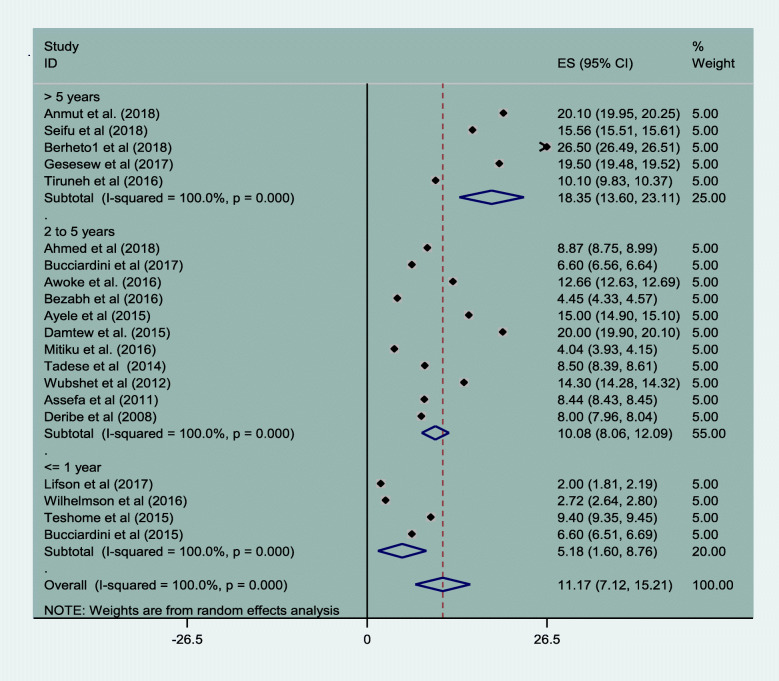
Magnitude of transfer out of HIV patients in Ethiopia from 2005 to 2019

About 11.17% (95% CI: 7.12, 15.21) of all study subjects transferred out during the study period. The cumulative transfer out percentage was lowest in the earliest years of follow up, and highest by the end of the follow-up period. 5.18% (95% CI: 1.60, 8.76) and 18.35% (95% CI: 13.60, 23.11) Fig. 7.

### Factors related to patient retention and attrition

#### Socio-demographic and behavioral factors

Five original papers stated that marital status was associated with patient retention in clinical care. Of which three articles reported that married people were less likely to be lost to follow up [8, 30, 51] and the probability of dropout for patients with separated marital status was about 16.82% higher than those patients with marital status divorced [41]. Another article [44] reported as single marital status as an independent predictor of death. Pooled odds ratio from 13 primary studies indicated that currently unmarried people are 1.52 times more likely not to be retained in clinical care (OR, 1.52, 95% CI, 1.15–2.01). Moreover, ten papers reported that male patients were less likely to be retained in HIV care in Ethiopia [8, 16, 23, 30, 33, 36, 53, 55, 59, 60]. Yet, one study in Ethiopia reported that females were less likely to be retained in clinical care [26]. However, it was not statically significant from pooled analysis of odds ratio from18 primary studies (OR, 1.12, 95% CI, 0.97–5.45). Similarly, eight papers [17, 22, 23, 28, 30, 36, 49, 54] reported that young age was an important predictor of loss to follow up but this was not statistically significant in the pooled analysis (OR, 0.88, 95% CI, 0.27–2.89). Four papers reported that educational status had an influence on retention in care [10, 30, 41, 54]. All except one [41] primary articles has reported that people with no or primary education were more likely to experience loss to follow up compared to those with secondary and above educational level. Two studies reported that urban residents were better in retention than their rural counterparts [22, 49]. Three papers indicated that economic constraints such as being dependent patients for source of food and daily laborers were risk factors for treatment interruption [10, 49, 51]. Being a merchant, farmer, daily labour and jobless had a greater risk of dropout [38, 41]. On the other hand, one study reported that merchants were at higher risk of death [45] However, except marital status, none of the socio-demographic factors were found statistically significant for patient retention in care (Table 3).

**Table 3:** Socio-demographic factors associated with HIV/AIDS patients’ attrition from care in Ethiopia (2005–2019)

Variables	No*	Sample	OR (95% CI)	I^2^ (%)	*P*-value
Age in years					
18–24	8	12,482	1	99	< 0.001
> 24			0.88 (0.27–2.89)		
Sex					
Female	18	33,424	1	82.1	< 0.001
Male			1.12 (0.97–5.45)		
Marital status					
married	13	18,911	1	90.8	< 0.001
Not married			1.52 (1.15–2.01)**		
Employment status					
Employed	3	1887	1	70	0.036
Not employed			1.27 (0.86–1.88)		
Residence					
Urban	2	12,735	1	93.3	< 0.001
Rural			0.87 (0.43–1.78)		
Education					
Educated	8	6016	1	96.7	< 0.001
Not educated			2.21 (0.90–5.45)		
Disclosure status					
Disclosed	2	2008	1	71.1	0.063
Not disclosed			6.36 (3.58–11.29)**		
Substance use					
Yes	2	815	1	75.5	0.044
No			0.41 (0.17–0.98)**		

No*---stands for numbers of articles

**Significant at *p*-value less than 0.05

HIV patients who did not disclose their HIV status were 6.36 times more likely to experience attrition from clinical care (OR, 6.36, 95% CI, 3.58–11.29). Patients who were not substance users were 59% more likely to be retained in clinical care (OR, 0.41, 95% CI, 0.17–0.98). Mental health issues such as taking hard drugs (cocaine, cannabis and IV drugs) and excessive alcohol drinking were risk factors for treatment defaulting [52]. Any types of substance use was reported as risk for loss to follow up [38]. It was also reported that patients with higher score for stress were poor in ART adherence [57]. Moreover, patients with mental status being not at ease were at increased risk for loss to follow up [51] (Table 3).

#### Clinical related factors

Fourteen studies [10, 18, 23, 24, 31, 35, 38, 43, 44, 54, 57–60] indicated that poor functional status (ambulatory or bed ridden) was a factor for patient attrition (OR, 2.11, 95% CI 1.33–3.34). Twelve papers [17, 18, 22, 23, 30, 43–45, 49, 54, 58, 59] reported that patients with advanced WHO clinical stages III or IV were also at greatest risk of death and loss to follow up (OR, 1.85, 95% CI, 1.36–2.51). Seventeen papers [8, 10, 27, 29–31, 35, 38, 43–45, 49, 55, 57–60] reported that baseline CD4 count less than 200 cells/ μL and greater than 350 cells/ μL was reported as risk factor for attrition but it was not significant in the pooled odds ratio analysis (OR, 1.09, 95% CI,0.49–2.41). Four papers reported that lower level of hemoglobin is a risk factor for death [18, 39, 43, 44]. Four studies reported that lower weight and BMI is risk factor for patient death and loss to follow up [38, 44, 45, 57].

Treatment related factors such as baseline ART regimen of Zidovudine-Lamivudine-Nevirapine (AZT-3TC-NVP) was key factor for patient attrition [22, 33]. Six studies [18, 22, 24, 38, 43, 57] demonstrated that poor drug adherence was a risk factor for patient attrition of both loss to follow up and death (OR, 6.60, 95% CI 1.41–30.97). Three studies reported that adverse drug side effects are responsible for patient loss to follow up and death [18, 39, 57]. Six studies reported that opportunistic infection were the main responsible factors for patient death and loss to follow up includes [38, 39]. These included TB [39, 44–46] and diarrhea for more than 3 months led a risk of death [18]. Nine papers reported that provision of isoniazid preventive therapy (IPT) [17, 18, 35, 38, 39, 50, 54] and CPT [25, 29] prophylaxis indicted that optimize patient retention.

Two papers reported on quality of care indicated that patients whose next appointment weren’t recorded were at risk of loss to follow up [8, 16]. Three papers reported that loss to follow up was higher among HIV patients in hospitals compared to health centers [28, 37, 54] while, one study reported that loss to follow up was higher among patients attending health centers than those in hospitals [53]. On the other hand, two studies reported contrary result one as higher death rate in health centers than in hospitals and other is vice versa [37, 54]. The pooled odds ratio indicated that there is no difference on level of retention based on health facility (OR, 1.23, 95% CI, 0.58–2.62) Table 4.

**Table 4:** Clinical factors associated with HIV/AIDS patients’ attrition from care in Ethiopia (2005–2019)

Variables	No*	Sample	OR (95% CI)	I^2^ (%)	*P*-value
ART adherence					
Good	5	6080	1	98.1	< 0.001
Poor/fair			6.60 (1.41–30.97)**		
Functional status					
Working	12	9954	1	92.1	< 0.001
Ambulatory/bedridden			2.11 (1.33–3.34)**		
Opportunistic disease					
Yes	8	7574	1	94.3	< 0.001
No			0.52 (0.30–0.90)**		
Recorded appointment					< 0.001
Yes	2	2518	1	97.6	
No			0.7 (0.17–2.88)		
BMI					
Normal (> 18.5)	4	2260	1	70.7	0.017
Underweight (< 18.5)			2.21 (1.45–3.39)**		
side effects					
Yes	2	1196	1	98.8	< 0.001
No			0.83 (0.03–26.98)		
INH					
Took INH	4	2275	1	97.7	< 0.001
Not took INH			3.42 (0.57–20.51)		
Health facility					
Health center	2	11,717	1	84.5	0.011
Hospital			1.23 (0.58–2.62)		
Baseline WHO stage					
I and II	12	21,509	1	92	< 0.001
III and IV			1.85 (1.36–2.51)**		
Baseline CD4 level					
< 200	8	19,134	1	98.3	< 0.001
> 200			1.09 (0.49–2.41)		
Hemoglobin					
Anemic (< 10 g/ml)	2	815	1	0	0.35
Normal (> 10 g/ml)			0.29 (0.20–0.42)**		

No*---stands for numbers of articles, ** significant at *p*-value < 0.05

## Discussion

This systematic review and meta-analysis investigated available evidence on the magnitude and associated factors of HIV patient retention and attrition in clinical care in Ethiopia.

The study indicated that the pooled prevalence of HIV patients’ clinical retention was 70.65%; and attritions was 15.17% loss to follow up, 6.75% death, and 11.17% transfer out. Furthermore, the incidence of loss to follow up was 13.79 person years of observation. Factors such as being currently unmarried, non-disclosed of HIV status, history of poor drug adherence, poor functional status, presence of opportunistic infections, lower BMI, substance use, lower hemoglobin and advanced WHO clinical stages were significantly associated with patient attrition.

Levels of HIV patient retention in antiretroviral treatment program in Ethiopia (70.65%) was comparable to the report from a study in India (70.7%) [64]. On the other hand, it was lower than patient retention in Asia (80%) [4], Sub Saharan Africa (77.5%) and Cape Town, South Africa 94% [65–67], Anambra, Nigeria (80%) [68], KwaZulu-Natal, South Africa (77.5%) [69], Khayelitsha, South Africa (85.9%) [5] and Southeastern United States (91.7%) [70]**.** This could be explained by the inclusion in our analysis of studies with longer follow-up periods. The longer the follow up period the lower the rates of clinical retention [69]. In addition, other possible reasons for the observed differences include the fact that countries have different levels of infrastructure, HIV burden, and HIV associated stigma that can cause loss to follow and transfer out to other health facilities [71].

On the other hand, the level of patient retention in this study is higher than that observed studies conducted among HIV infected patients from Africa (65%), Latin America and Caribbean (64%) [4], Asia-Pacific Region [72], Tanzania (25%) [73] and South Africa [74]. In Africa, Asia, and Latin America that the value ranged from 3.1 to 45.1% [75]. The reason might be the differences in cut off point for loss to follow up (LTFU) and treatment eligibility criteria of the latter two studies. Now a days, immediate initiation of ART following HIV positive test result may contributed for good patient retention [66, 74, 76–78]. This might be due to test and treat strategy is effective in patient retention since it decreases the chance of loss to follow up from determining treatment eligibility to adherence preparation [79, 80].

In this study, rate of HIV patients lost to follow up, death and transfer out is similar with study report form India [64] but higher than study from Australia and Asia [81]. Loss to follow up was the major cause of attrition, followed by death and transfer out. This is in line with a previous systematic review in sub-Saharan Africa [67].

On the other hand, rate of lost to follow up (13.79 per 100 person years of observation) was lower than observed from previous studies from Asia Pacific region (21.4 person years of observation) [72], Malawi (26 and 48 per 100 person years of observation for pre-ART and ART patients respectively), Guinea-Bissau (51.1 per 100 person-years of observation) [82] and study conducted among children in Ethiopia 29.7% [7]. The reason can be due to difference of study population and the current study was composite of both earlier and recent times study findings while the previous study was conducted at the beginning of the treatment. Moreover patient retention was better in Asia than Africa from previous study [61] that reflects country specific difference in terms of patients retention. Moreover, the pooled death rate was 6.75% which is lower than previous systematic review 5–40% of death in Ethiopia [83]. The variation may be due to small sample size in the previous study and different time of follow up.

Our review identified a number of socio-demographic and clinical factors that could be targets for future interventions. It is indicated that currently unmarried people (never married, separated, divorced and widowed) were 1.52 times more likely not to be retained in care. This is similar with a study in South Africa that reported women without regular partner were not retained in clinical care [69]. This might be because of married people get support from their spouse to adhere to their treatment. Likewise, those who did not disclose their HIV status to someone were 6.36 times more likely not to be retained in care. This can be explained by the fact that those who disclosed their sero-status can get support from someone who knew the HIV status and this facilitates regular treatment attendance. This might be due to people who had perceived HIV related stigma are more likely to experience treatment attrition [13] . Therefore, partner testing, HIV status disclosure and mutual support are important components of HIV intervention [3, 84]. In contrast to these findings, one study in Nigeria showed that married women were poor in clinical retention [68]. These differences may be due to difference by cultural practices and the attitude of people on the role of gender in both countries.

Substance abuse and mental distress have negative effects on HIV patient retention in clinical care. This finding is in line with a systematic review in developing countries [85]. This might be due to poor drug adherence as a result of distressed mental status. Retention in care was significantly higher in patients treated by mental health trained healthcare workers [23]. This can be explained by the fact that the decision making ability of patients abusing substances is altered and this facilitates poor treatment adherence [13]. Inline to this, the previous studies also reported that patients with no bereavement concern were less likely to experience loss to follow up while patients with mental status not at ease were at increased risk of defaulting from treatment [50, 51].

It was evidenced that advanced clinical stage such as poor baseline functional status, WHO clinical stage III or IV, suboptimal ART adherence, presence of one or more opportunistic infections and being underweight had higher risk of patient attrition. Such findings are similar to findings from studies in Ethiopia [83], Malawi ([86], Guinea-Bissau [82] and Asia Pacific region [72]. The reasons for patients to be non-adherent were forgetfulness, side effects, feeling sick and running out of medication [87].

### Limitation of the study

Original articles used different length of follow up period that had variable outcomes of attrition. Moreover, studies conducted across different treatment guidelines revision may affect patient retention and treatment outcome.

## Conclusion

About two third of HIV patients were retained in care. The most common cause of attrition among HIV positive people in Ethiopia were loss to follow up, transfer out and death respectively. It can be concluded that Ethiopia has long to walk to achieve the minimum targets of patient retention achieved by most of low income countries. There were increased attrition rate for longer period of follow up that need due attention from clinicians. Due to change of treatment eligibility criteria from time to time and varying definitions of loss to follow up, it is necessary to reconsider the definitions of LTFU. Moreover, tracing studies are necessary to rule out final destination of loss to follow up patients. Social support is necessary for all HIV patients and encouraging disclosure to family members. This also implied HIV patients require mental health interventions in addition to the medical model of treatment. Patients with poor drug adherence, advanced HIV disease stage, opportunistic infection, poor functional status (ambulatory or bedridden), underweight and anemic patients need special attention that includes adequate adherence preparation and nutritional intervention. In general it was evidenced that number of socio-demographic determinants prone patients for loss to follow up while the clinical factors leads to death. Hence, in order to achieve the desired level of patient retention in clinical care, a comprehensive interventions which are targeted at socio-demographic, clinical, laboratory and behavioral factors is necessary.

## Supplementary information

**Additional file 1: Table S1.** Quality assessment of articles for attrition of HIV positive people in care and its determinants in Ethiopia (2005–2019).

## Data Availability

Data from this manuscript is available upon request by contacting the corresponding author.

## Abbreviations

AIDS: Acquired Immunodeficiency Syndrome
CI: Confidence Interval
HIV: Human immunodeficiency virus
LTF: Loss to Follow up
WHO: World Health Organization
