# Supplementary material for: HIV patients retention and attrition in care and their determinants in Ethiopia: a systematic review and meta-analysis
Source: BMC Infect Dis. 2020 Jun 22;20:439. doi: 10.1186/s12879-020-05168-3 (PMC7310275; doi:10.1186/s12879-020-05168-3)
Supplement: Supplementary file 1 — Additional file 1: Table S1. Quality assessment of articles for attrition of HIV positive people in care and its determinants in Ethiopia (2005–2019). [file 12879_2020_5168_MOESM1_ESM.docx]

**Additional file 1**

**Table 1: Quality assessment of articles for attrition of HIV positive people in care and its determinants in Ethiopia (2005-2019)**

| Serial number | Author | Years of publication | **Score of 1 or 0 ((Yes=1, No=1, Unclear(UN)=0, Non applicable (NA)=0)** | | | | | | | | | | | Reasons for zero score |
| --- | --- | --- | --- | --- | --- | --- | --- | --- | --- | --- | --- | --- | --- | --- |
|  |  |  | 1 | 2 | 3 | 4 | 5 | 6 | 7 | 8 | 9 | total | status |  |
|  | Anmut et al. | 2018 | 1 | 1 | 1 | 1 | 1 | 1 | 1 | 1 | 1 | 9 | selected |  |
|  | Yigzaw et al | 2018 | 1 | 1 | 1 | 1 | 1 | 1 | 1 | 1 | 1 | 9 | selected |  |
|  | Seifu et al | 2018 | 1 | 1 | 1 | 1 | 1 | 1 | 1 | 1 | 1 | 9 | selected |  |
|  | Berheto1 et al | 2018 | 1 | 1 | 1 | 1 | 1 | 1 | 0 | 1 | 1 | 8 | selected | Exposed and unexposed is changed, but the outcome is measured correctly. |
|  | Assemie et al | 2018 | 1 | 1 | 1 | 1 | 1 | 1 | 1 | 1 | 1 | 9 | selected |  |
|  | Adewo et al | 2018 | 1 | 1 | 1 | 1 | 1 | 1 | 1 | 1 | 1 | 9 | selected |  |
|  | Gesesew et al | 2017 | 1 | 1 | 1 | 1 | 1 | 0 | 1 | 1 | 1 | 8 | selected | The definition of ART discontinuation was broad and not specific |
|  | Bucciardini et al | 2017 | 1 | 1 | 1 | 1 | 1 | 1 | 1 | 1 | 1 | 9 | selected |  |
|  | Wilhelmson et al | 2016 | 0 | 0 | 1 | 1 | 1 | 1 | 1 | 1 | 1 | 7 | selected | Both adults and children were mixed up |
|  | Tiruneh et al | 2016 | 1 | 1 | 1 | 1 | 1 | 1 | 1 | 1 | 1 | 9 | selected |  |
|  | Megerso et al | 2016 | 1 | 1 | 1 | 1 | 1 | 1 | 1 | 1 | 1 | 9 | selected |  |
|  | Mitiku et al. | 2016 | 1 | 1 | 1 | 1 | 1 | 1 | 1 | 1 | 1 | 9 | selected |  |
|  | Teshome et al | 2015 | 1 | 1 | 1 | 1 | 1 | 1 | 1 | 1 | 1 | 9 | selected |  |
|  | Desalegn et al | 2015 | 1 | 1 | 1 | 1 | 1 | 1 | 1 | 1 | 1 | 9 | selected |  |
|  | Melaku et al | 2015 | 1 | 1 | 1 | 1 | 1 | 1 | 1 | 1 | 1 | 9 | selected |  |
|  | Shaweno et al | 2015 | 1 | 1 | 1 | 1 | 1 | 1 | 1 | 1 | 1 | 9 | selected |  |
|  | Mekuria et al | 2015 | 1 | 1 | 1 | 1 | 1 | 1 | 1 | 1 | 1 | 9 | selected |  |
|  | Bucciardini et al | 2015 | 1 | 1 | 1 | 1 | 1 | 1 | 1 | 1 | 1 | 9 | selected |  |
|  | Assefa et al | 2015 | 1 | 1 | 1 | 1 | 1 | 1 | 1 | 1 | 1 | 9 | selected |  |
|  | Berheto et al | 2014 | 1 | 1 | 1 | 1 | 1 | 1 | 1 | 1 | 1 | 9 | selected |  |
|  | Reepalu et al | 2014 | 1 | 1 | 1 | 1 | 1 | 1 | 1 | 1 | 1 | 9 | selected |  |
|  | Tadesse et al | 2014 | 1 | 1 | 1 | 1 | 1 | 1 | 1 | 1 | 1 | 9 | selected |  |
|  | Wubshet et al | 2013 | 0 | 0 | 1 | 1 | 1 | 1 | 1 | 1 | 1 | 7 | selected | The objective of the primary study was different from the systematic review |
|  | Asefa et al | 2013 | 1 | 1 | 1 | 1 | 1 | 1 | 1 | 1 | 1 | 9 | selected |  |
|  | Ahmed et al | 2013 | 1 | 1 | 1 | 1 | 1 | 1 | 1 | 1 | 1 | 9 | selected |  |
|  | Wubshet et al | 2012 | 1 | 1 | 1 | 1 | 1 | 1 | 1 | 1 | 1 | 9 | selected |  |
|  | Assefa et al | 2011 | 1 | 1 | 1 | 1 | 1 | 1 | 1 | 1 | 1 | 9 | selected |  |
|  | Balch et al | 2010 | 1 | 1 | 1 | 1 | 1 | 1 | 1 | 1 | 1 | 9 | selected |  |
|  | Deribe et al | 2008 | 1 | 1 | 1 | 1 | 1 | 1 | 1 | 1 | 1 | 9 | selected |  |
|  | Abebe et al | 2014 | 1 | 1 | 1 | 1 | 0 | 1 | 1 | 1 | 1 | 8 | selected | The outcome variable was death |
|  | Ahmed et al | 2018 | 1 | 1 | 1 | 1 | 1 | 1 | 1 | 1 | 1 | 9 | selected |  |
|  | Mekonnen et al | 2019 | 1 | 1 | 1 | 1 | 1 | 1 | 1 | 1 | 1 | 9 | selected |  |
|  | Chaka et al. | 2019 | 1 | 1 | 1 | 1 | 1 | 1 | 1 | 1 | 1 | 9 | selected |  |
|  | Tadege | 2018 | 1 | 1 | 1 | 1 | 1 | 1 | 1 | 1 | 1 | 9 | selected |  |
|  | Gezea et al. | 2019 | 1 | 1 | 1 | 1 | 1 | 1 | 1 | 1 | 1 | 9 | selected |  |
|  | Mekonnen et al. | 2018 | 1 | 1 | 1 | 1 | 1 | 1 | 1 | 1 | 1 | 9 | selected |  |
|  | Damitew et al | 2015 | 1 | 1 | 1 | 1 | 1 | 1 | 1 | 1 | 1 | 9 | selected |  |
|  | Ayele et al | 2015 | 1 | 1 | 1 | 1 | 1 | 1 | 1 | 1 | 1 | 9 | selected |  |
|  | Bezabh et al | 2016 | 1 | 1 | 1 | 1 | 1 | 1 | 1 | 1 | 1 | 9 | selected |  |
|  | Awoke et al | 2016 | 1 | 1 | 1 | 1 | 1 | 1 | 1 | 1 | 1 | 9 | selected |  |
|  | Mekuria et al | 2016 | 1 | 1 | 1 | 1 | 1 | 1 | 1 | 1 | 1 | 9 | selected |  |
|  | Assefa et al | 2017 | 1 | 1 | 1 | 1 | 1 | 1 | 1 | 1 | 1 | 9 | selected |  |
|  | Lifson et al | 2017 | 1 | 1 | 1 | 1 | 1 | 1 | 1 | 1 | 1 | 9 | selected |  |
|  | Tadege | 2018 | 1 | 1 | 1 | 1 | 1 | 1 | 1 | 1 | 1 | 9 | selected |  |
|  | Telele et al | 2018 | 1 | 1 | 1 | 1 | 1 | 1 | 1 | 1 | 1 | 9 | selected |  |

**Key: corresponding horizontal values are assigned as follows;**

1. Was the sample frame appropriate to address the target population?
2. Were study participants sampled in appropriate way?
3. Was the sample size adequate?
4. Were the study subjects and the setting described in detail?
5. Was the data analysis conducted with sufficient coverage of the identified sample?
6. Were valid methods used for the identification of the condition?
7. Was the condition measured in a standard, reliable way for all participants?
8. Was there appropriate statistical analysis?
9. And was the response rate adequate, and if not, was the low response rate managed appropriately

- **Values for quality assessment against the 9 criteria**
- Yes=1
- No=0
- Unclear=0
- Not applicable=1
